# Resistance of Nile tilapia fed with *Padina boergesenii* extract to *Pseudomonas putida* infection

**DOI:** 10.1186/s12917-024-04115-7

**Published:** 2024-06-29

**Authors:** Karima A. Bakry, Mahmoud Nasr, Zeinab Al-Amgad, Ereen kondos, Malak K. N. Kondos, Pierre E. Mehanny, Abdullah A. A. Alghamdi, Mohsen A. Khormi, Hanan H. Abd-ElHafeez, Walaa F.A. Emeish

**Affiliations:** 1 Department of Fish Diseases, Faculty of Veterinary Medicine, South Valley University, Qena, 83523 Egypt; 2 General Authority for Veterinary Services, Qena Veterinary Directorate, Qena, Egypt; 3 Department of Pharmacology, Faculty of Veterinary Medicine, South Valley University, Qena, 83523 Egypt; 4 National Institute of Oceanography and Fisheries (NIOF), Cairo, 11516 Egypt; 5 Department of Biochemistry, Toxicology and Feed Deficiency, Agriculture Research Center (ARC), Animal Health Research Institute (AHRI), Giza, 12618 Egypt; 6 Department of Biology, Faculty of Science, Al-Baha University, Al-Baha, Saudi Arabia; 7 Department of Biology, College of Science, Jazan University, P.O. Box. 114, Jazan, 45142 Kingdom of Saudi Arabia; 8 Department of Cell and Tissues, Faculty of Veterinary Medicine, Assiut University, Assiut, 71526 Egypt

**Keywords:** Brown algae, *Oreochromis niloticus*, GC-MS analysis, Serum biochemical parameters, Oxidative stress, Gene expression, Bacterial challenge

## Abstract

The aim of this research was to estimate the immunopotentiation effect of brown algae *Padina boergesenii* water extract on Nile tilapia, *Oreochromis niloticus* through resistance to *Pseudomonas putida* infection. Gas Chromatography Mass Spectrometry was utilized to characterize the seaweed phytoconstituents. One hundred and twenty-six fish were divided in triplicates into two equal groups corresponding to two diet variants that used to feed Nile tilapia for 20 successive days: a basal (control), and *P. boergesenii* water extract supplemented group. Fish samples were collected at 10-days intervals throughout the experiment. Serum biochemical constituents, total antioxidant capacity (TAC), and some immune related genes expression of the spleen and intestinal tissues of experimental fish were studied, as well as histological examination of fish immune tissues. Moreover, following 20 days of feeding, the susceptibility of Nile tilapia to *P. putida* infection was evaluated to assess the protective effect of the used extract. The findings indicated that the studied parameters were significantly increased, and the best immune response profiles were observed in fish fed *P. boergesenii* water extract for 20 successive days. A bacterial challenge experiment using *P. putida* resulted in higher survival within the supplemented fish group than the control. Thus, the lowered post-challenge mortality of the fish may be related to the protection provided by the stimulation of the innate immune system, reduced oxidative stress by higher activity of TAC, and elevated levels of expression of iterleukin-1beta (*IL-1β*), beta-defensin (*β-defensin*), and natural killer-lysin (*NKl*). Moreover, the constituents of the extract used showed potential protective activity for histological features of the supplemented fish group when compared to the control. Collectively, this study presents a great insight on the protective role of *P. boergesenii* water extract as an additive in Nile tilapia feed which suggests its potential for improving the immune response against *P. putida* infection.

## Introduction

Nile tilapia, *O. niloticus*, aquaculture has experienced a substantial expansion in Egypt and other tropical nations [1]. Nevertheless, the extensive growth of their population is confronted with various infections and specific strategies for handling them that have the potential to harm the well-being of both humans and animals [2]. Disease outbreaks significantly hinder the long-term viability of Nile tilapia aquaculture production. *Pseudomonas* and other bacterial septicemia provide the greatest risk to Nile tilapia in Egypt [3]. Previous research has indicated that *P. putida* is a significant bacterial pathogen that impacts a range of fish species [4, 5], resulting in elevated mortality rates and substantial economic loss [6]. *P. putida* was recorded from Nile tilapia in Egypt and resulted in exophthalmia, ascites, and ulcers on the fish body [5, 7].

Different chemotherapeutic agents can be employed to prevent various illnesses in aquaculture. Nevertheless, their utilization is accompanied by antibiotic resistance against bacteria [8], antibiotic residues in tissues [9], and pollution of the aquatic environment [10]. Therefore, there is a growing demand for new approaches to manage bacterial infections [11] and there is an increasing interest in using creative management approaches to enhance aquatic welfare and ensure the construction of safe food [12]. These alternate measures involve the utilization of natural bioactive substances that activate the immune system of fish.

Seaweeds are inexpensive and readily accessible sources of bioactive chemicals that can be utilized as immunostimulants in aquaculture [13]. The seaweed *P. boergesenii* is a type of brown algae, that is frequently encountered in coastal areas worldwide. *P. boergesenii* has lately gained considerable attention in aquaculture due to its diverse range of active principles that possess highly powerful immunostimulant, antioxidant, and antibacterial characteristics [14, 15]. A recent study indicated that consuming *P. boergesenii* in fish diet may have the ability to combat *Pseudomonas aeruginosa* infection [16].

Serum biochemical and innate immune markers are employed to assess the impact of feed additives on fish [17, 18]. Recently, there has been a growing focus on studying the alterations in the expression of genes relevant to the immune system. IL-1β plays a crucial role in the inflammatory response in fish [19] and is a key component of the innate immune mechanism [20]. It can activate lymphocytes and other cytokines, which in turn can stimulate phagocytic cells.

Antimicrobial peptides are constituents of the fish’s innate immune system which offer defense against bacteria, and other pathogens [21, 22]. Many AMPs inhibit bacterial colonization by employing either a lytic or ionophoric mechanism [23]. β-defensin and NKL are two prominent and widely recognized groups of antimicrobial peptides (AMPs). β-defensin exhibits potent antibacterial activity against infections that affect fish [24, 25]. Furthermore, the transcriptome expression of this antimicrobial peptide (AMP) was found to be elevated in fish that were given immunostimulants [26]. NKL is synthesized by cytotoxic T lymphocytes (CTLs) and natural killer (NK) cells and exhibits a wide spectrum of activity against many microbial infections [27]. Hence, the target of this work was to investigate the immunomodulatory impacts of *P. boergesenii* water extract when added to Nile tilapia feed. Serum biochemical indicators, some immune gene expression response, and the histological architecture of the intestine, kidney, liver, and spleen will be evaluated in this study. Furthermore, a challenge experiment will be carried out to evaluate the disease resistance capabilities of Nile tilapia against *P. putida* infection.

## Materials and methods

### Collection and identification of brown algae

Samples from seaweed *P. boergesenii*, a species of brown algae, was collected from the coastal region of the Red Sea near Hurghada city during the summer of 2022. The National Institute of Oceanography and Fisheries (NIOF) gathered and identified the seaweed material. Following the collection process, the algae were promptly rinsed with seawater to eliminate any attached substances and contaminants, such as sand particles and epiphytes, and then sterilized using distilled water. According to Allender and Kraft [28] macroalgae species were taxonomically identified based on their morphology. Samples were left to dry at room temperature, crushed into fine powder, and then sieved following the method of Cho et al. and Gonzalez et al. [29, 30].

### Preparation of *P. boergesenii* water extract

The brown alga, *P. boergesenii* (Phaeophyceae, Dictyotaceae) watery extract was prepared according to Ghaednia et al. [31]. Briefly, 20 g of powdered sea algae were dissolved in 300 ml of distilled water in a round bottom flask and boiled for about 3 h at 100 °C, then filtrated using filter paper, and the filtered extract was lyophilized using a rotary evaporator till obtain solid watery extract.

### Gas chromatography-mass spectrometry (GC/MS) analysis of *P. Boergesenii*

The *P. boergesenii* extract underwent analysis using GC-MS at the National Research Center in Dokki, Cairo, Egypt. The specimen was introduced into an HP-5 column (30 m × 0.25µM film thickness) for GC–MS analysis. The PerkinElmer Clarus 580/560 S model system was utilized to convey the gas at a flux rate of 0.8 mL/min. The temperature of the gas chromatography (GC) oven was set to increase at a rate of 2 °C/min., starting from 60 °C and reaching 250 °C. The total ion current is determined by calculating the relative area values of the volatile composition as a proportion of the total.

### Diets preparation

The basal diet used in this experiment was purchased from Skretting Company of Animal Nutrition, Egypt, and contained 30% crude protein, 9.5% ash, 6% crude lipid, and 5.22% crude fiber. The control diet, which served as the basal diet, was not supplemented with *P. boergesenii* water extract. Feeding rate used in this study was chosen according to the earlier finding in *Cirrhinus mrigala* [16] to detect the most suitable concentration of the *P. boergesenii* water extract powder. Hence, the supplemented diet was prepared by thoroughly mixing the basal diet with 4.5% concentration of *P. boergesenii* water extract powder until stiff dough was obtained, and water was used as the binding agent. The dough was prepared into pellets with the help of a hand pelletizer and then cut into small-sized pieces. The pellets were left to dry at room temperature and were preserved in plastic containers at -20^o^C until use.

### Fish samples and experimental design

The methodology and fish experiments undertaken in this study were approved by the Ethical Committee of the Faculty of Veterinary Medicine, Assiut University, Assiut, Egypt. These experiments adhered to the regulations set by the OIE guidelines for the use of animals in research, under the approval No. 06/2024/0179.

A total of 150 live, healthy, cultured Nile tilapia, with an initial average body weight of 65 ± 6 g, were collected from the Nag Hamady fish hatchery in Qena Governorate. They were transported in oxygenated tanks to the Animal Experiment Lab, Fish Diseases Division, Faculty of Veterinary Medicine, South Valley University.

The fish were acclimated to laboratory conditions for a period of 14 days in fully equipped 500 L fiberglass tanks. During this time, they were fed commercial pellets without any additional substances, twice a day, at a quantity equivalent to 3% of their body weight. The tanks were provided with dechlorinated tape water containing a salinity of 0.2 parts per thousand (ppt), and the duration of light and darkness was regulated to 12 h each. A total of ten Nile tilapia specimens were randomly captured and subsequently tested negative for *P. putida* using specific primer (Table 1) in polymerase chain reaction which give 380 bp (bp).

**Table 1:** List of primer sequences used in the present study

Gene	Primer name	Nucleotide sequence (5’-3’)	References
*Pseudomonas putida*	*P. putida*	F: CCAAAACTGGCAAGCTAGAGTAC R: CATCTCTGGAAAGTTCTCTGC	[65]
Beta -defensin	*β-defensin*	F: GGTTGTTTTGGCACTTTTGGTT R: TGTTGGGAGGCAAACCTTTCT	[66]
Natural killer-lysin	*NKL*	F: ATTTGCGGCACAGTGATTT R: ATGGAAGTCTTGATGGGGCT	[49]
Interleukin-1 Beta	*IL-1β*	F: TGCACTGTCACTGACAGCCAA R: ATGTTCAGGTGCACTATGCGG	[67]
Elongation factor 1-α	*EF1α*	F: CTACGTGACCATCATTGATGCC R: AACACCAGCAGCAACGATCA	[68]
Beta actin	*β -actin*	F: CAGCAAGCAGGAGTACGATGAG R: TGTGTGGTGTGTGGTTGTTTTG	[69]

Feeding trials for this study were set up and continued for 20 days in three replicates, where acclimated Nile tilapia (*n =* 126) was randomly divided into 2 equal groups (control and supplemented) that represent the two nutritional experiments (corresponding to the two diet variants). Each group was divided into two subgroups, including 18 (*n =* 6 per replicate) and 45 (*n =* 5 per replicate) fish, used for sampling (sampling tanks) to evaluate the serum parameters, histological examination, and gene expression analysis of fish, and for the challenge experiment (challenge tanks), respectively.

During the feeding period, the experimental diets were given to the fish twice a day, at a dosage of 3% of their body weight. With dissolved oxygen at 7 ± 0.5 mg /L, pH at 7 ± 0.4, and water temperature at 25 ± 0.5^o^C, the tank water was kept in an ideal condition.

### Blood and tissue sampling

Blood samples were obtained from the experimental fish (*n =* 2/group/replicate) at the 10th and 20th days of the feeding period. The fish were randomly caught and anesthetized with 0.05 mL/L clove oil [32]. Blood was collected from the caudal vein by using blood collection tubes. Samples were left for 3–4 h at room temperature to coagulate, then centrifuged at 4000 rpm for 25 min. The serum was collected and stored at − 20^o^C until used for further analysis.

Fish were euthanized with 0.5 mL/L of clove oil after blood sampling, and then the spleen and intestine were immediately collected (*n =* 1/group/replicate) and placed in RNAlater (Qiagen) at 4 °C overnight and then stored at − 20 °C until the further analysis.

### Analysis of serum biochemical indices

The serum analysis was conducted utilizing commercially available test kits as per the manufacturer’s recommended procedures. The parameters examined were total protein and albumin (Biomed Diagnostics, Badr city, Egypt). The results were determined through colorimetric analysis in a T80 Spectrophotometer (PG Instrument, Leicestershire, UK). To get the globulin level (g/dL), the albumin value is subtracted from the total protein value using a mathematical calculation [33]. The TAC level was assessed after the method of Koracevic et al. [34], utilizing commercially accessible test kits from Bio diagnostic, located in Dokki, Egypt.

### Challenge experiment with *P. Putida*

The pathogenic *P. putida* strain used for the challenge study was isolated from Nile tilapia, *O niloticus*, showing typical signs of septicemia during an outbreak in a local commercial farm. The strain was identified by various biochemical characters as *pseudomonas* species and confirmed molecularly as *P. putida* using 16s rDNA gene sequencing (GenBank accession no. OM048106, 838 bp). The strain isolated was identified and preserved at -80 °C.

*Pseudomonas putida* colonies were recovered by cultivating them on pseudomonas agar base and incubating them at a temperature of 28^o^C for 24 h. Subsequently, one colony was selected and cultured into 100 mL of tryptic soy broth and then incubated at a temperature of 28^o^C for one night. The bacterial suspension utilized for infecting Nile tilapia was quantified by assessing the optical density by spectrophotometric absorbance at a wavelength of 600 nm, as well as by determining the colony-forming unit (cfu) counts using a standard plate-count method [35].

To restore virulence, the *P. putida* was administered 3 passages to Nile tilapia via intra-peritoneal injection (500 µL broth culture) before to the actual challenge experiment. Colony morphology and biochemical profile allowed for the probable identification of *P. putida* after its isolation from the kidneys of injected Nile tilapia. Molecular analysis with primers specific to *P. putida* validated the identification **(**Table 1**).**

In the challenge subgroups (control and supplemented), 3 tanks (*n =* 15) were set for clinical signs and mortality records (mortality tanks), another 3 tanks (*n =* 15) were used for post-challenge gene expression analysis and histological examination (challenge sampling tanks) and additional 3 tanks (*n =* 15) were assigned as bacterial challenge negative control group.

After 20 days of feeding, fish of the challenge tanks (mortality and challenge sampling tanks) from both feeding groups (*n =* 30/group) were removed from their tanks using a net, anesthetized, and subjected to a 500 µL intraperitoneal injection of *P. putida* at a concentration of 1 × 10^6^ cfu/mL. The negative challenge control group (*n =* 15) received an intraperitoneal injection of a 500 µL sterile physiological saline solution. The mortality rate was observed and documented daily for a period of 14 days post-challenge. In addition, samples of moribund and recently dead fish were taken from the kidney to verify *P. putida* presence as the reason of the documented mortality and observed clinical symptoms. The results were stated as percentage of mortality. After 2 days of post-challenge, 3 fish from each feeding group (1 fish / sampling tank) were randomly chosen and euthanized. The spleen and gut were sampled to obtain total RNA.

### Gene expression analysis

#### Total RNA extraction and cDNA synthesis

The tissue was disrupted and homogenized, and total RNA was extracted from the spleen and intestinal tissues using the RNeasy® Mini kit (Qiagen, Germany) according to the manufacturer’s instructions. The RNA was eluted from the column and its purity and concentration were assessed using a nano-photometer spectrophotometer (NanoDrop™ LITE Spectrophotometer, Thermo Scientific, USA). For cDNA synthesis, 1 µg of total RNA from each sample (with a purity of 2.1 ± 0.2) was reverse transcribed using the RevertAid first strand cDNA synthesis kit (Thermo Scientific, USA) according to the manufacturer’s instructions. Subsequently, the first cDNA products were preserved at a temperature of -20 °C until used for the amplification in the quantitative real-time PCR (qRT-PCR).

### Target genes and primers

To analyze the expression of our target immune genes, we evaluated the expressions of *IL*-*1β, β-defensin*, and *NKL* genes using qRT-PCR. The primer sequences utilized in the gene expression study are provided in Table 1. The relative expression was normalized using the Nile tilapia beta-actin (*β-actin*) and elongation factor 1-alpha *(EF1α)* genes as reference genes.

### Expression of the target genes and data visualization

The HERA^PLUS^ SYBR® Green Master Mix (Willowfort, England) was utilized to perform qRT-PCR, following the instructions provided by the manufacturer. The reaction consisted of 10 µL of 2× SYBR® Green, 1 µL of each primer for the target gene, and 2 µL of cDNA template. The reaction was conducted in a total volume of 20 µL, which was adjusted to 20 µL by adding PCR grade sterile nuclease free water. The thermal cycling protocol for *IL-1β, β-defensin*, *β-actin*, and *EF1α* genes consisted of an initial denaturation step at 95 °C for 3 min. to activate the DNA polymerase, followed by 40 cycles of denaturation at 95 °C for 30 s, combined annealing, and extension at 60 °C for 1 min., and a final extension at 95 °C for 10 s. The thermal cycling protocol for *NKL* involved an initial denaturation phase at 95 °C for 3 min., followed by 40 cycles of denaturation at 95 °C for 30 s., annealing at 58 °C for 30 s., and extension at 72 °C for 30 s., and a final extension step at 95 °C for 10 s. Fluorescent data was gathered during the extension process. No template control was included in all reactions. The thermal protocol for the melting curve analysis was conducted at the end of the amplification process, with a temperature range of 65℃ to 95℃ with increase by 0.5℃ every 0.05 s., and the cycle threshold (Ct) value was determined. The qRT-PCR tests were conducted using the CFX96™ Real-Time PCR detection system manufactured by BIO-RAD in the United States. The calculation of gene expression for the target genes was performed using the delta-delta Ct (2^−ΔΔCt^) method as described by Livak and Schmittgen [36].

### Histological examination

Liver, anterior kidney, spleen, and intestinal samples were obtained from fish (2/replicate/group) at the 10th and 20th days of the feeding period; moreover, the same tissue samples were collected post-challenge from the challenge sampling tanks. The tissues were transferred to be fixed in 10% buffered formalin for histological examination. The fixed tissues undergo classical histological procedures through consecutive dehydration in the varying series of ethanol solution, clearing in xylene and consequently embedding in paraffin. Stained hematoxylin and eosin (H&E) sections were prepared for histomorphology identification [37].

### Statistical analysis

The data analysis and figure construction were done using GraphPad Prism software (version 9.3.0, San Diego, CA, USA). The data were reported as the mean ± standard error of the mean (SEM). Statistical analysis was performed using a One-way analysis of variance test (ANOVA) to compare the two experimental groups. Differences were judged significant when the (*P* > 0.05). Subsequently, Tukey’s Post-Hoc test was employed to do multiple comparisons.

## Results

### Identification of the brown algae

The collected seaweed samples were identified as brown algae: *P. boergesenii* (Phaeophyceae, Dictyotaceae). The taxonomic identification of the seaweed is based on their morphological and anatomical characters.

### Gas Chromatography-Mass Spectrometry (GC/MS) analysis

**Fig. 1:**
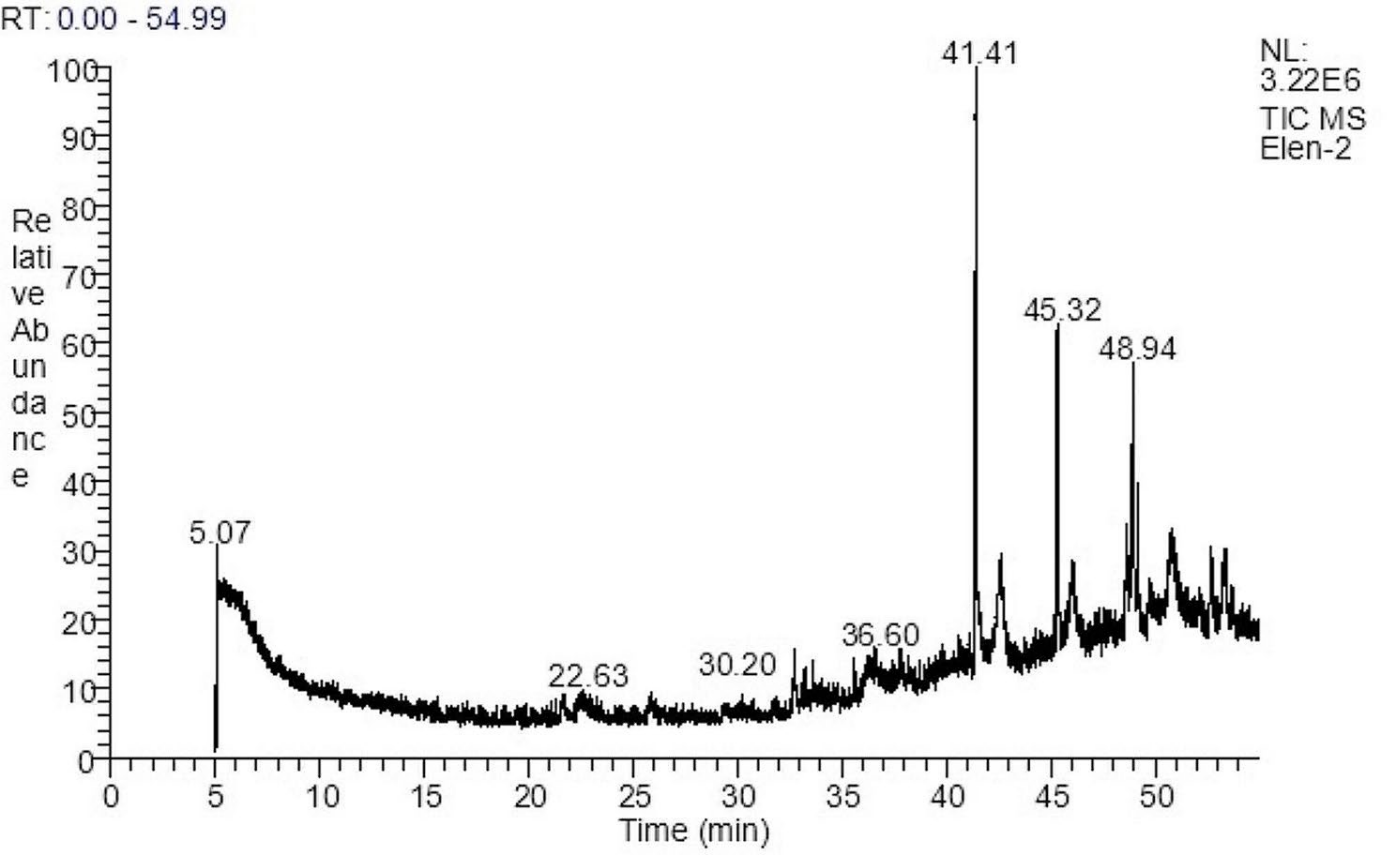
Chromatogram obtained from the GC/MS of the watery extract of *P. boergesenii*

The GC–MS chromatogram (Fig. 1) showed various compounds present in *P. boergesenii* water extract. The biological activity and the major compounds with their retention time (RT) and peak area (%) are listed in Table 2.

**Table 2:** Biological activity, molecular weight, and formula of GC/MS analysis of *Padina boergesenii*

Retention Time (min.)	Molecular weight	Molecular formula	Peak area (%)	Name of the compound	Biological activity
5.09	128	C_2_H_2_Cl_2_O_2_	5.90	Acetic acid, Dichloroacetaldehyde	Antimicrobial [70]
32.72	308	C_20_H_36_O_2_	2.12	Z, Z-4,16 Octadecadien-1-ol acetate	antimicrobial and cytotoxic [71]
33.23	282	C_18_H_34_O_2_	1.06	9-Octadecenoic acid (Z)- (CAS)	Antibacterial, antioxidant, and anti-inflammatory [72]
35	446	C_28_H_46_O_4_	2.19	Octadecanoic acid, (4-4-yl) methyl ester	Cytotoxicity, antioxidant, and anti-inflammatory [73, 74]
41.41	282	C_18_H_34_O_2_	14.50	2-Propenoic acid, pentadecyl ester	anticancer, and cytotoxic [75]
42.62	122	C_69_H_98_O_6_	4.04	Docosahexaenoic acid, 1,2,3-propanetriyl ester	Cytotoxic, and anti-proliferative [76]
45.32	278	C_16_H_22_O_4_	10.14	1,2-Benzenedicarboxylic acid mono (2-ethylhexyl) ester	antioxidant and anti-proliferative [52] and antimicrobial and anticancer [53]
45.32	187	C_10_H_9_N_3_O	10.14	1’,2’,4’-Triazol-1’-yl	Antibacterial activity [77]
46	691	C_37_H_74_NO_8_P	3.22	Hexadecanoic acid	Antibacterial [55]
48.65	366	C_26_H_54_	4.73	Octadecane, 3-ethyl-5-(2-ethylbutyl)	antimicrobial [78]
48.65	496	C_32_H_64_O_3_	4.73	Palmitic acid, 2-(tetradecyloxy) ethyl ester	Anticancer [79] Antibacterial, antifungal, and antioxidant [54, 79]
48.94	310	C_22_H_46_	8.16	Docosane	Antimicrobial [80]
48.94	618	C_44_H_90_	8.16	Tetratetracontane	antibacterial [81] Potent anticancer and antimicrobial [82]
48.94	408	C_29_H_60_	8.16	Nonacosane (CAS)	Antimicrobial, strong anti-inflammatory, and antioxidant [83, 84]
49.20	182	C_11_H_18_O_2_	3.07	1,6-Octadien-3-ol, 3,7-dimethyl-, formate	Anti-inflammatory and anti-cancer [85]
52.15	594	C_27_H_30_O_15_	1.58	Flavone	Anti-inflammatory and antioxidant [86]

### Serum biochemical parameters

The levels of total protein, albumin, globulin, and A: G ratio were cited in Table 3. It was found that the serum total protein and globulin levels were not significantly different between the supplemented and control groups on the 10th day of feeding, but both were increased in the supplemented group on the 20th day. The serum albumin level was not statistically different between the supplemented and control groups during the feeding experiment. The A: G ratio in the serum of the supplemented group was reduced significantly on the 20th day of feeding. The activity of TAC was significantly increased in the supplemented group (Table 3) during the time course of dietary supplementation with *P. boergesenii* water extract.

**Table 3:** Effect of feeding diet supplemented with *P. boergesenii* water extract for 10 or 20 days on Nile tilapia serum biochemical parameters

Parameters	Experimental days	Control	Supplemented group
**Total protein (g/dl)**	10 20	5.226 ± 0.26 ^a^ 4.91 ± 0.19 ^a^	5.914 ± 0.28 ^a^ 6.54 ± 0.25 ^b^
**Albumin (g/dl)**	10 20	1.79 ± 0.27 ^a^ 1.83 ± 0.11 ^a^	1.42 ± 0.16 ^a^ 1.78 ± 0.14 ^a^
**Globulin (g/dl)**	10 20	3.44 ± 0.31 ^a^ 3.07 ± 0.27 ^a^	4.5 ± 0.41 ^a^ 4.76 ± 0.13 ^b^
**A/G ratio**	10 20	0.57 ± 0.12 ^a^ 0.63 ± 0.09 ^a^	0.34 ± 0.07 ^a^ 0.37 ± 0.02 ^b^
**Total antioxidant capacity**	10 20	0.67 ± 0.12 ^a^ 0.67 ± 0.08 ^a^	1.3 ± 0.12 ^b^ 1.4 ± 0.04 ^b^

The data are presented as Means ± Standard error (*n =* 6). Different letters in the same raw indicate significant difference at *P* < 0.05

### Challenge experiment

**Fig. 2:**
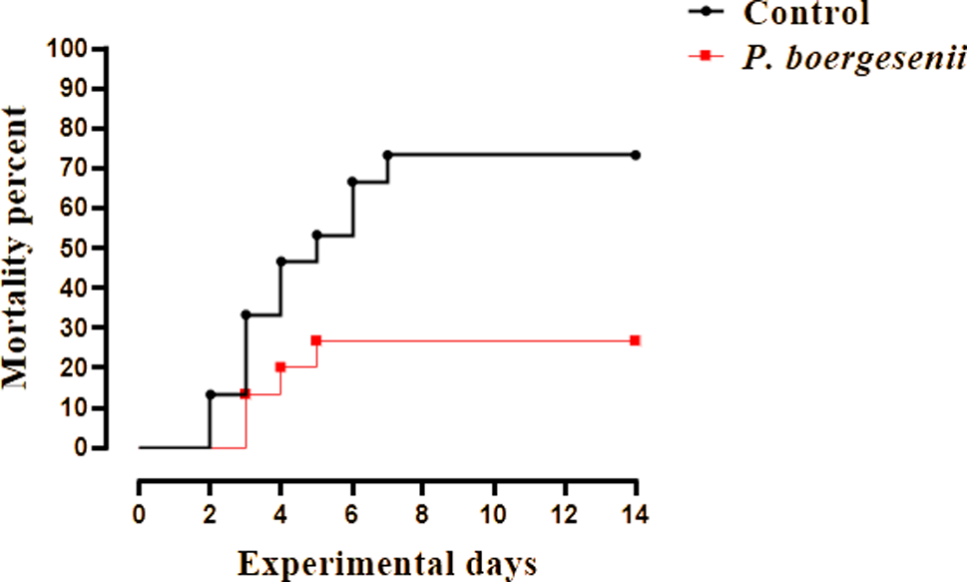
Cumulative mortality percent of fish experimental groups challenged with *P. putida*

The results were presented in terms of percentage mortality (Fig. 2). The fish group injected with *P. putida* and fed on basal diet with no additives (control) revealed a total mortality rate of 73.33% within 14 days. They exhibited skin hemorrhages and skin ulceration, fins rot, dark pigmentation, detached scales, abdominal distention and tail and fins rot. Results of re-isolation of bacteria and their identification revealed the same characteristics of the injected bacteria.

In the fish group fed on a diet supplemented with *P. boergesenii* water extract, a mortality of 26.7% was recorded and less sever clinical signs and lesions were observed during the challenge experiment. Whereas neither clinical signs nor mortality were observed during the experiment in the negative challenge control group.

### Tissue distribution, and real-time PCR analysis of the expression levels of *IL-1β*, *β-defensin* and *NKL*

**Fig. 3:**
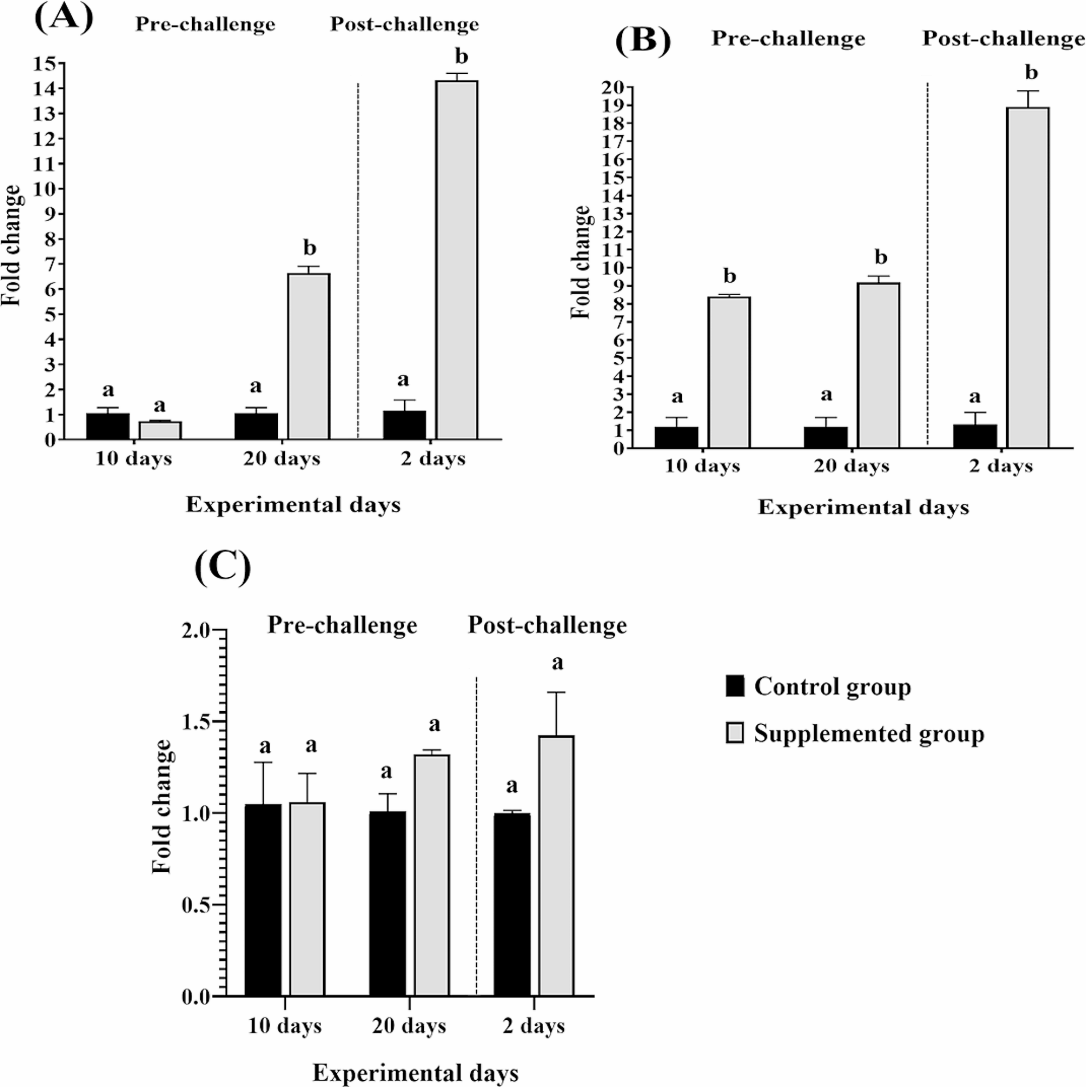
Gene expression profiles of *IL-1β* (**A**), *β-defensin* (**B**) and *NKL* (**C**) in the spleen of Nile tilapia following 10 and 20 days of feeding diet supplemented with 4.5% *P. boergesenii* water extract and 2 days post-challenge with *P. putida*. Data are presented as mean ± SEM (*n =* 3). The mean values denoted with different letters within the same experimental days are statistically significant (*P* < 0.05)

In this study, expression of the genes under study were statically evaluated in the supplemented group in comparison with their expression levels in the control group. There was a variation in the tissue level expression of *IL-1β*, *β-defensin*, and *NKL* in Nile tilapia fed with *P. boergesenii* water extract. *IL-1β* expression in the spleen of the supplemented group showed a significant upregulation on the 20th day of feeding of the experimental diet (Fig. 3A). While dietary supplementation with *P. boergesenii* had no effect on the *β-defensin* mRNA expression in the spleen of Nile tilapia (Fig. 3B). On the other hand, the expression of *NKL* gene in the spleen was upregulated during the time course of dietary supplementation with *P. boergesenii* water extract (Fig. 3C).

**Fig. 4:**
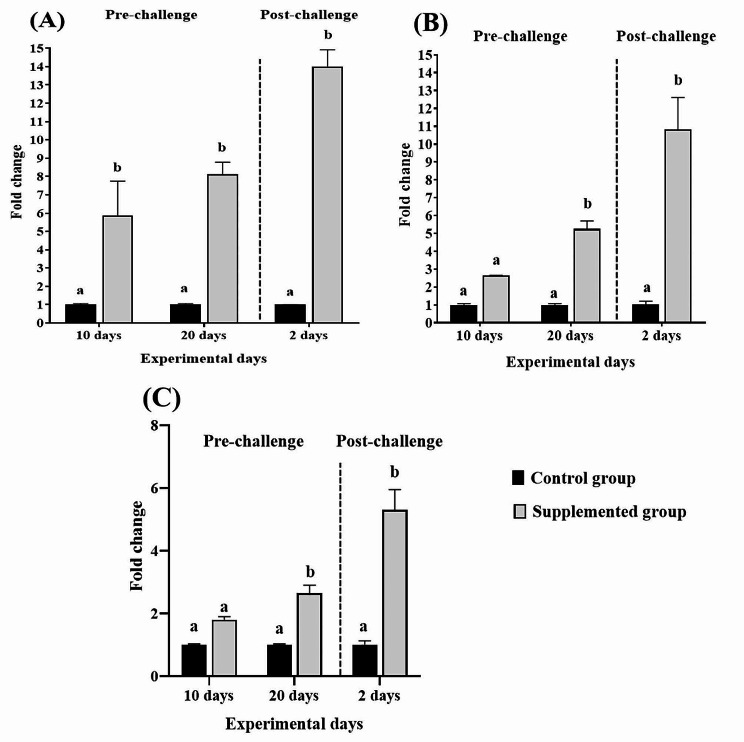
Gene expression profiles of *IL-1β* (**A**), *β-defensin* (**B**) and *NKL* (**C**) in the intestine of Nile tilapia following 10 and 20 days of feeding diet supplemented with 4.5% *P. boergesenii* water extract and 2 days post-challenge with *P. putida*. Data are presented as mean ± SEM (*n =* 3). The mean values denoted with different letters within the same experimental days are statistically significant (*P* < 0.05)

In addition, our study exhibited an upregulation in the *IL-1β* gene expression level in the intestine of the supplemented group during the feeding period (Fig. 4A). Whereas both AMPs (*β-defensin* and *NKL*) expression significantly upregulated in the intestine on the 20th day of feeding of the experimental diet (Fig. 4B & C).

Changes in the level of expression of the analyzed immune-related genes were also observed in the spleen and intestine after the *P. putida* challenge (Figs. 3 and 4), where all genes were significantly upregulated in both the spleen and intestine except the *β-defensin* expression in the spleen.

### Histological findings

#### Pre-challenge fish

##### Macroscopic results

Liver, anterior kidney, spleen, and intestine of the control and supplemented fish groups seemed to appear healthy without injury.

### Microscopic results

**Fig. 5:**
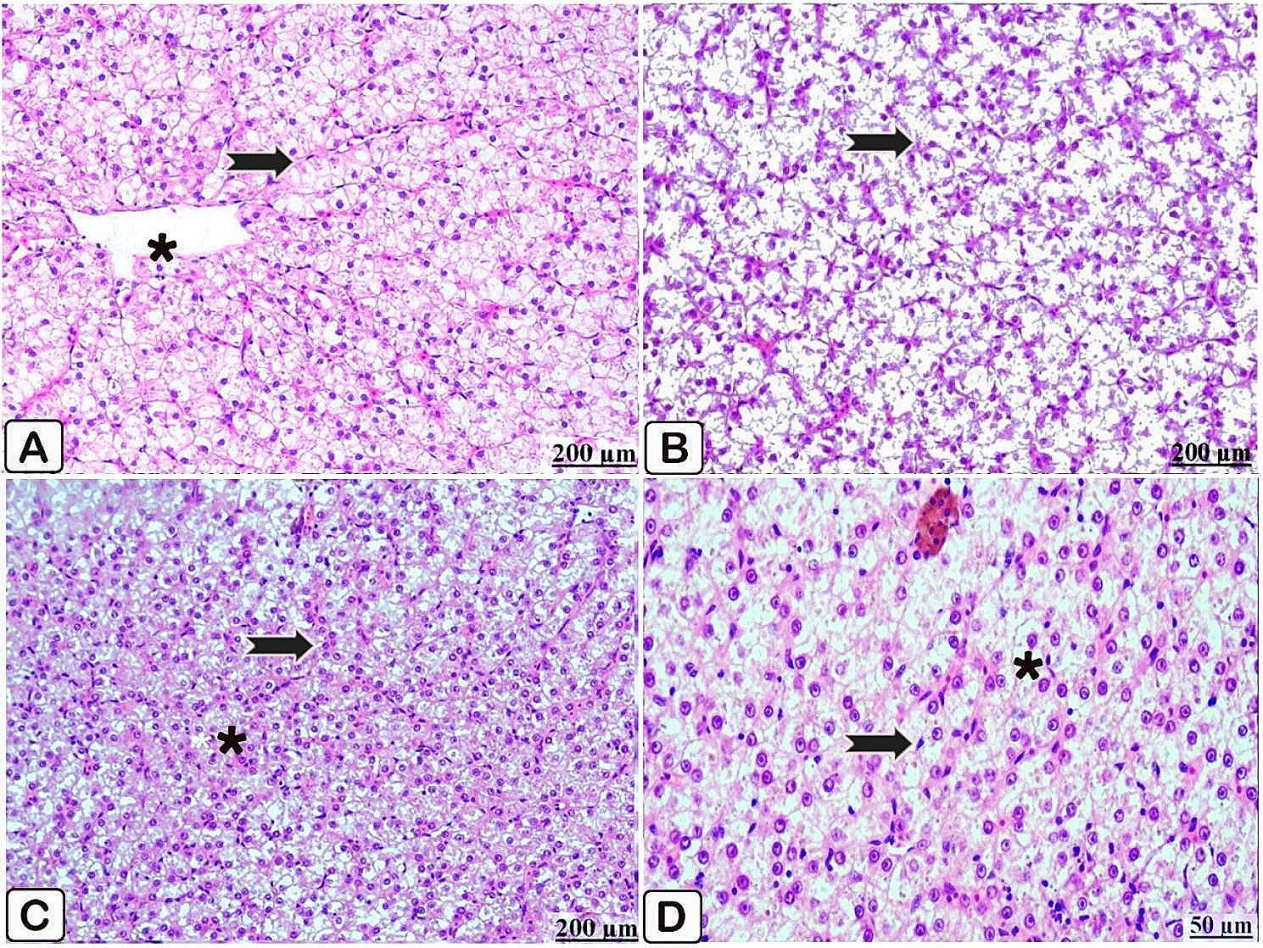
(**A**→**D**): Light microscopic sections with H &E stain of liver of control Nile tilapia (**A, B**), Nile tilapia fed diet supplemented with *P. boergesenii* water extract for 10 days (**C**) and Nile tilapia fed diet supplemented with *P. boergesenii* water extract for 20 days (**D**). (**A**) regular hepatocytes (arrow) and intact central vein (star). (**B**) intact hepatic cords (arrow). (**C**) well organized hepatic cords (arrow) and well identified kupffer cells (star). **(D**) significant detection of kupffer cells (arrow) beside this uniformly arranged hepatocytes (star)

Microscopic examination of the liver of the control fish revealed apparently healthy histomorphological structures where it exhibits uniform hepatic parenchyma (Fig. 5A, B). The liver of fish fed *P. boergesenii* water extract for 10 days manifested symmetrical hepatocytes and activation of kupffer cells (Fig. 5C). Likewise, after 20 days of supplementation, the liver showed regular hepatocytes (Fig. 5D).

**Fig. 6:**
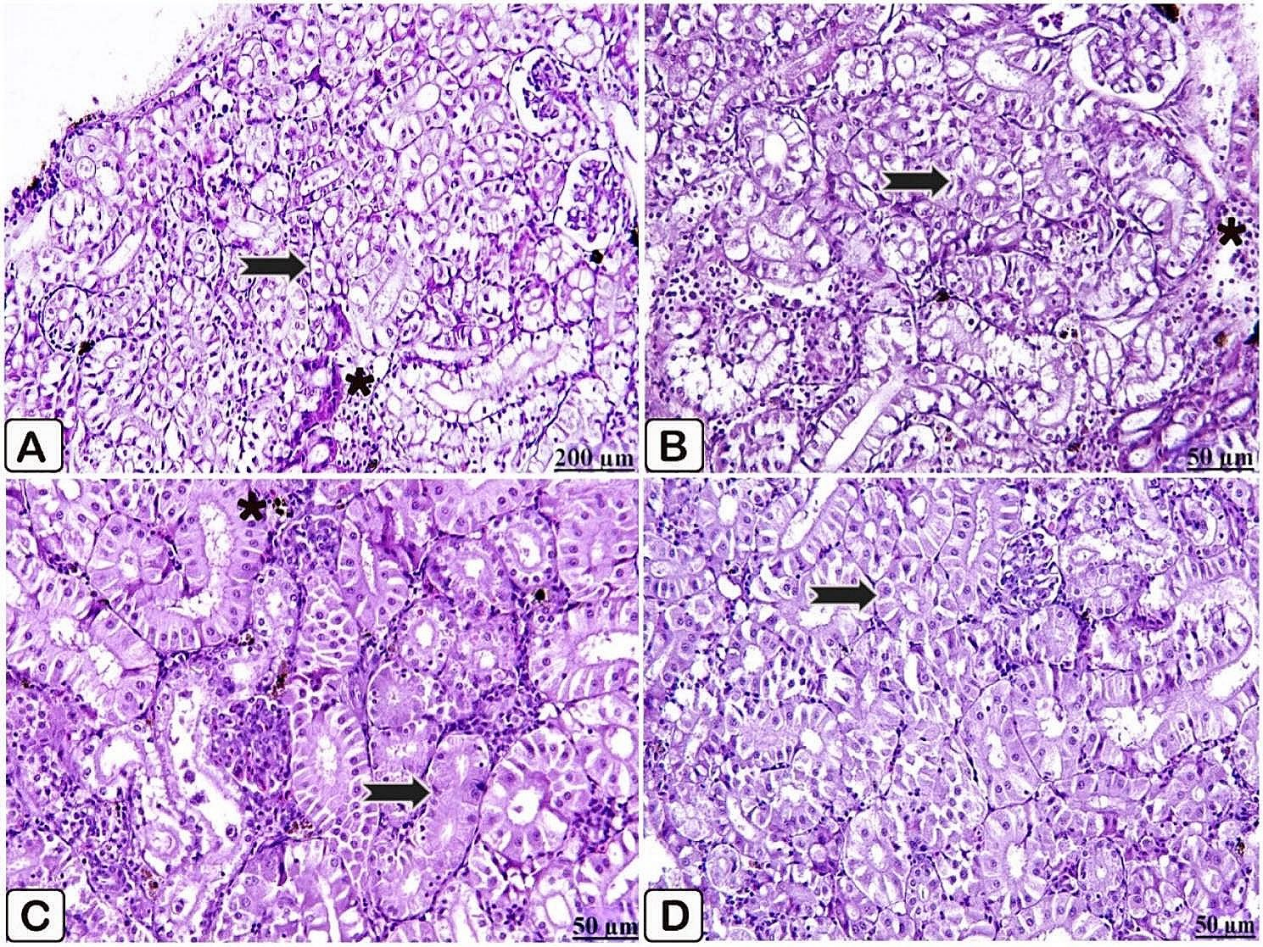
(**A**→**D**): Light paraffin microscopic sections with H &E stain of anterior kidney of control Nile tilapia (**A, B**), Nile tilapia fed diet supplemented with *P. boergesenii* water extract for 10 days (**C**) and Nile tilapia fed diet supplemented with *P. boergesenii* water extract for 20 days (**D**). **(A**) well-identified tubules (arrow) and normal appearance of hematopoietic tissues (star). (**B**) apparently healthy nephritic tubules (arrow), besides minimal lymphocytes aggregations (star). (**C**) normal nephritic tubules (arrow), and melanomacrophages infiltration (star). (**D**) well organized nephritic tubules (arrow)

Microscopic examination of the anterior kidney of the control fish illustrated normal nephritic tubules with minimally aggregated lymphocytes (Fig. 6A, B). Fish fed a diet supplemented with *P. boergesenii* water extract for 10 days provoked consistent architectures of the anterior kidney. Accordingly, kidney tissues were normally arranged in tubules with deeply infiltrated melanomacrophages (Fig. 6C). Simultaneously, regarding feeding with *P. boergesenii* for 20 days, the kidney showed regularly distributed tubules; besides this, a discrete distribution of melanomacrophages was observed (Fig. 6D).

**Fig. 7:**
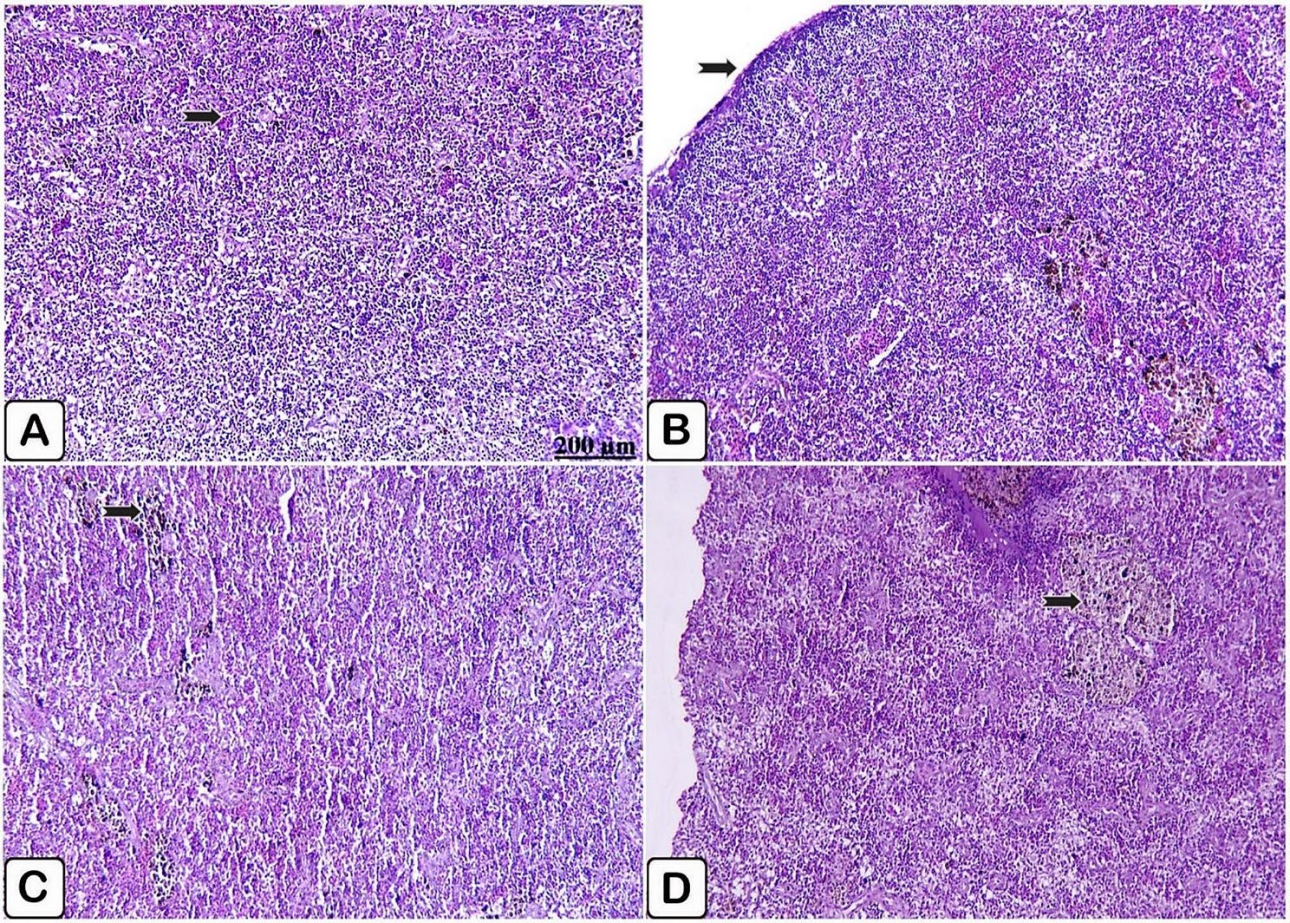
(**A**→**D**): Light paraffin microscopic sections with H &E stain of spleen of control Nile tilapia (**A**, **B**), Nile tilapia fed diet supplemented with *P. boergesenii* water extract for 10 days (**C**) and Nile tilapia fed diet supplemented with *P. boergesenii* water extract for 20 days (**D**). (**A**) normally arranged splenic parenchyma (arrow). (**B**) intact splenic capsules (arrow). (**C**) discrete distribution of melanomacrophages cells (arrow). (**D**) huge aggregation of melanomacrophages centers (arrow)

Microscopic examination of the spleen of the control fish exhibited splenic parenchyma in normal histological status (Fig. 7A, B). While the spleen of the supplemented group exhibited evidence of sharp activation of melanomacrophage cells either for a period of 10- or 20-days supplementation (Fig. 7C, D, respectively).

**Fig. 8:**
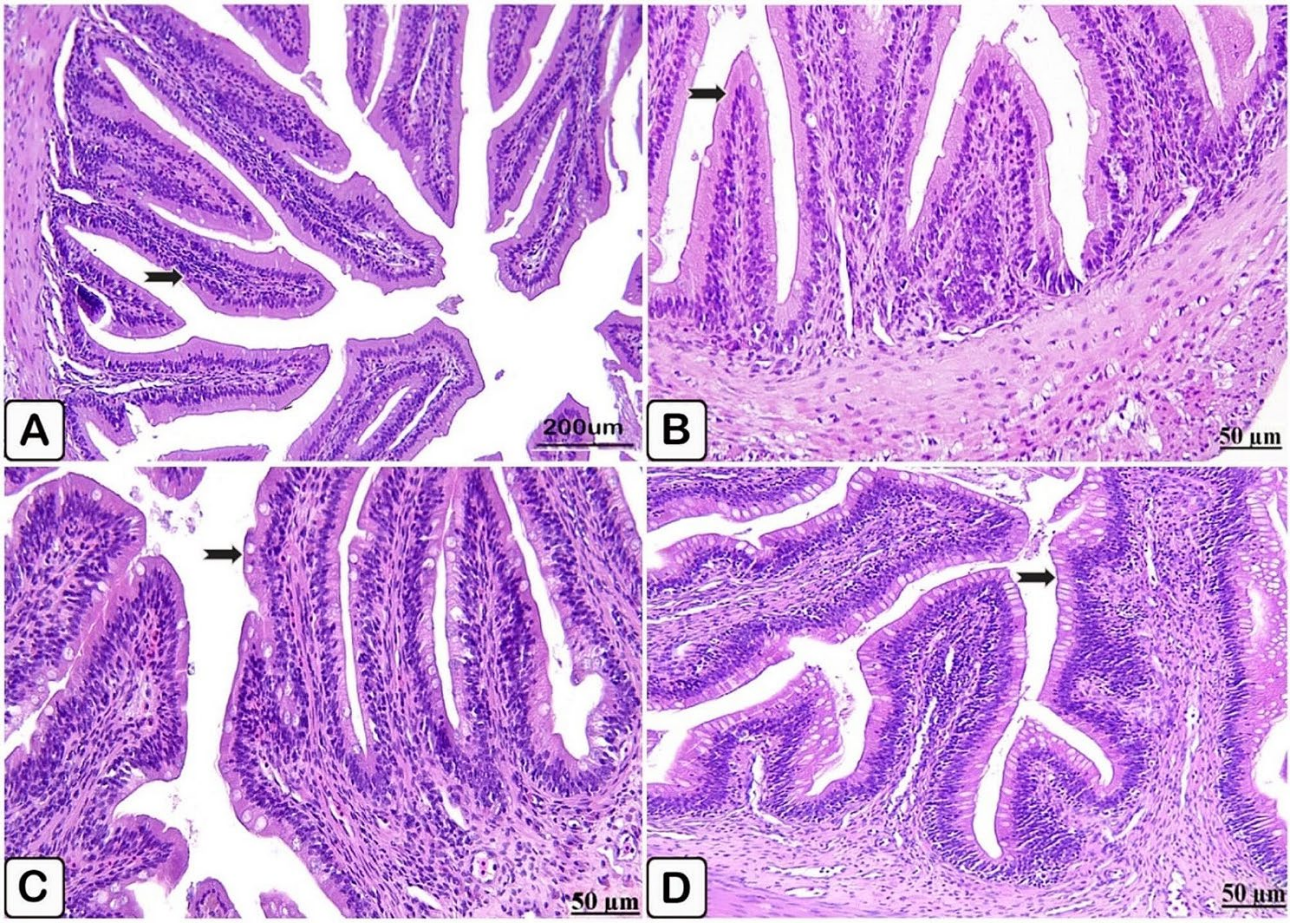
(**A**→**D**): Light paraffin microscopic sections with H &E stain of intestine of control Nile tilapia (**A, B**), Nile tilapia fed diet supplemented with *P. boergesenii* water extract for 10 days (**C**) and Nile tilapia fed diet supplemented with *P. boergesenii* water extract for 20 days (**D**). (**A**) normal epithelium lined intestinal villi (arrow). (**B**) less recognized goblet cells (arrow). (**C**) intact intestinal villi with increased goblet cells (arrow). (**D**) hyperactivation of goblet cells (arrow)

Microscopic examination of the intestine of the control fish revealed normally organized mucosa and submucosa layers, as well lack of goblet cells identification (Fig. 8A, B). In parallel to the control intestine histology, fish supplemented with *P. boergesenii* for 10 and 20 days demonstrated a good appearance of intestinal villi with increased goblet cells activation (Fig. 8C, D, respectively).

#### Post-challenge fish

##### Macroscopic results

Grossly, the organs under study, comprising the liver, anterior kidney, spleen, and intestine of the challenged supplemented group showed a good criterion. Meanwhile, the organs of the challenged control fish appeared distorted.

#### Microscopic results

**Fig. 9:**
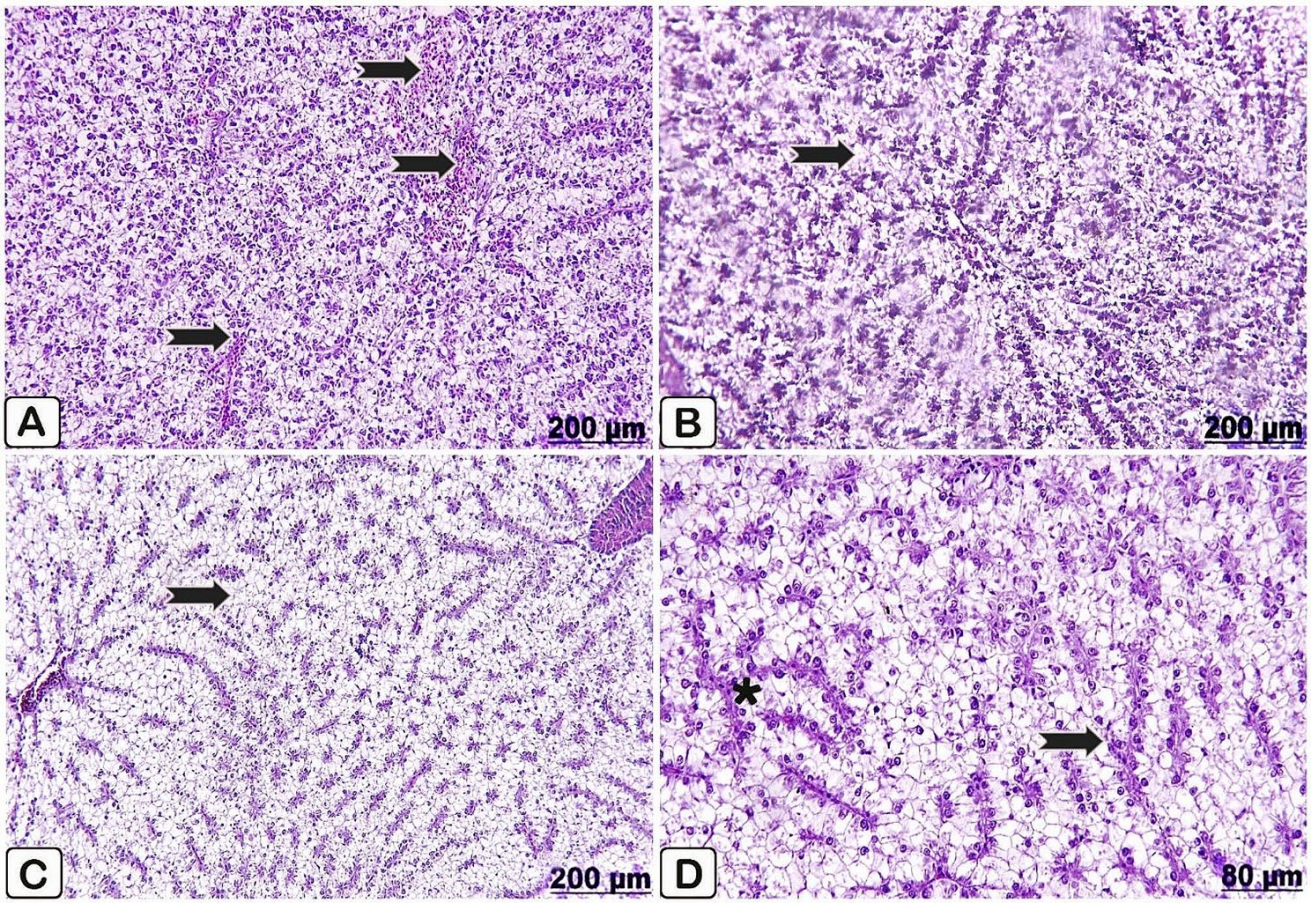
(**A**→**D**): Light paraffin microscopic sections with H &E stain from liver of control Nile tilapia challenged with *P. putida* (**A, B**), liver of Nile tilapia challenged supplemented group (**C, D**). (**A**) Light section showing congestion in the central vein (arrow). (**B**) Light section showing necrosis with vacuolization of the hepatocytes (arrow). (**C**) Light section showing mild hepatic vacuolation (arrow). (**D**) Light section showing activation of the kpuffer cells (arrow), and slightly congested blood sinusoids (star)

**Fig. 10:**
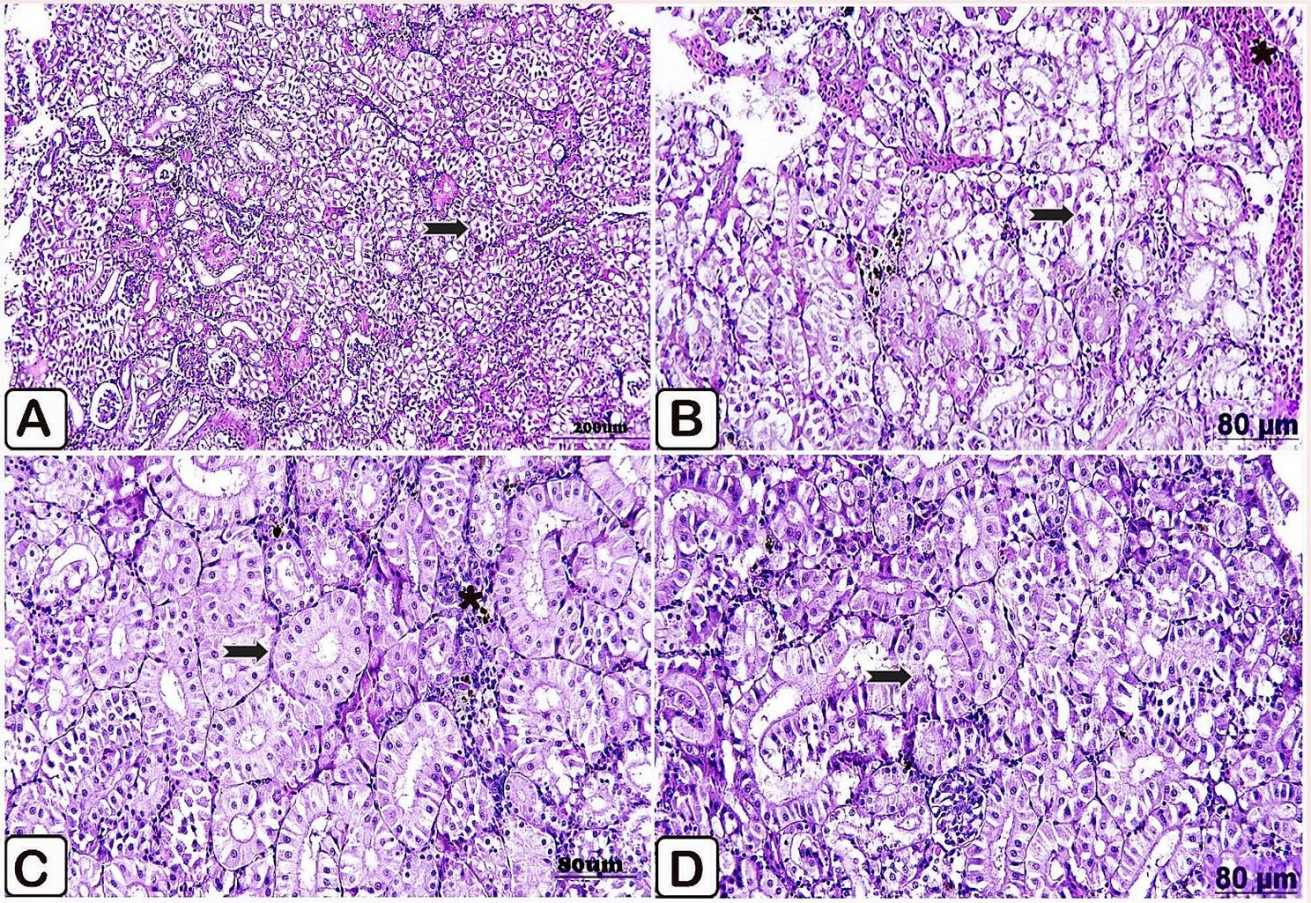
(**A**→**D**): Light paraffin microscopic sections with H &E stain from anterior kidney of control Nile tilapia challenged with *P. putida* (**A**, **B**), anterior kidney of Nile tilapia challenged supplemented group (**C**, **D**). A) Light section showing necrosis of the nephritic tubules (arrow). (**B**) Light section showing necrosis with cytoplasmic vacuolation of the renal tubular epithelium (arrow), besides congestion in the blood vessels (star). (**C**) Light section showing regular nephritic tubules (arrow) with normal hematopoietic tissues (star). (**D**) Light section showing normal kidney histoarchitecture (arrow)

**Fig. 11:**
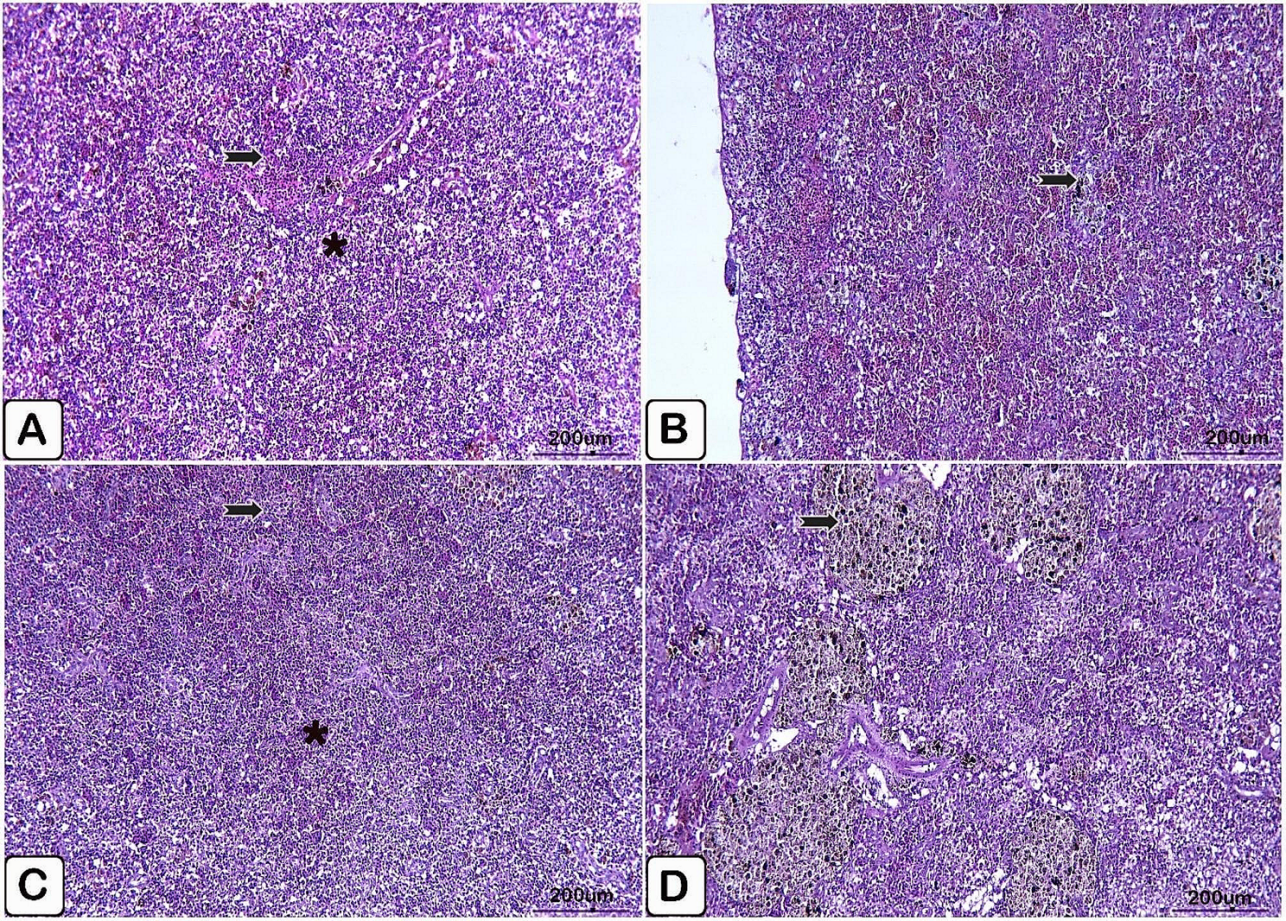
(**A**→**D**): Light paraffin microscopic sections with H &E stain from spleen of control Nile tilapia challenged with *P. putida* (**A, B**), spleen of Nile tilapia challenged supplemented group (**C, D**). (**A**) Light section showing congestion of the red pulp (arrow), moreover vacuolation of white pulp (star). (**B**) Light section showing minimal infiltration of melanomacrophages (arrow). (**C**) Light section showing intact white (arrow) and red (star) pulps. (**D**) Light section showing huge accumulation of melanomacrophages centers (arrow)

**Fig. 12:**
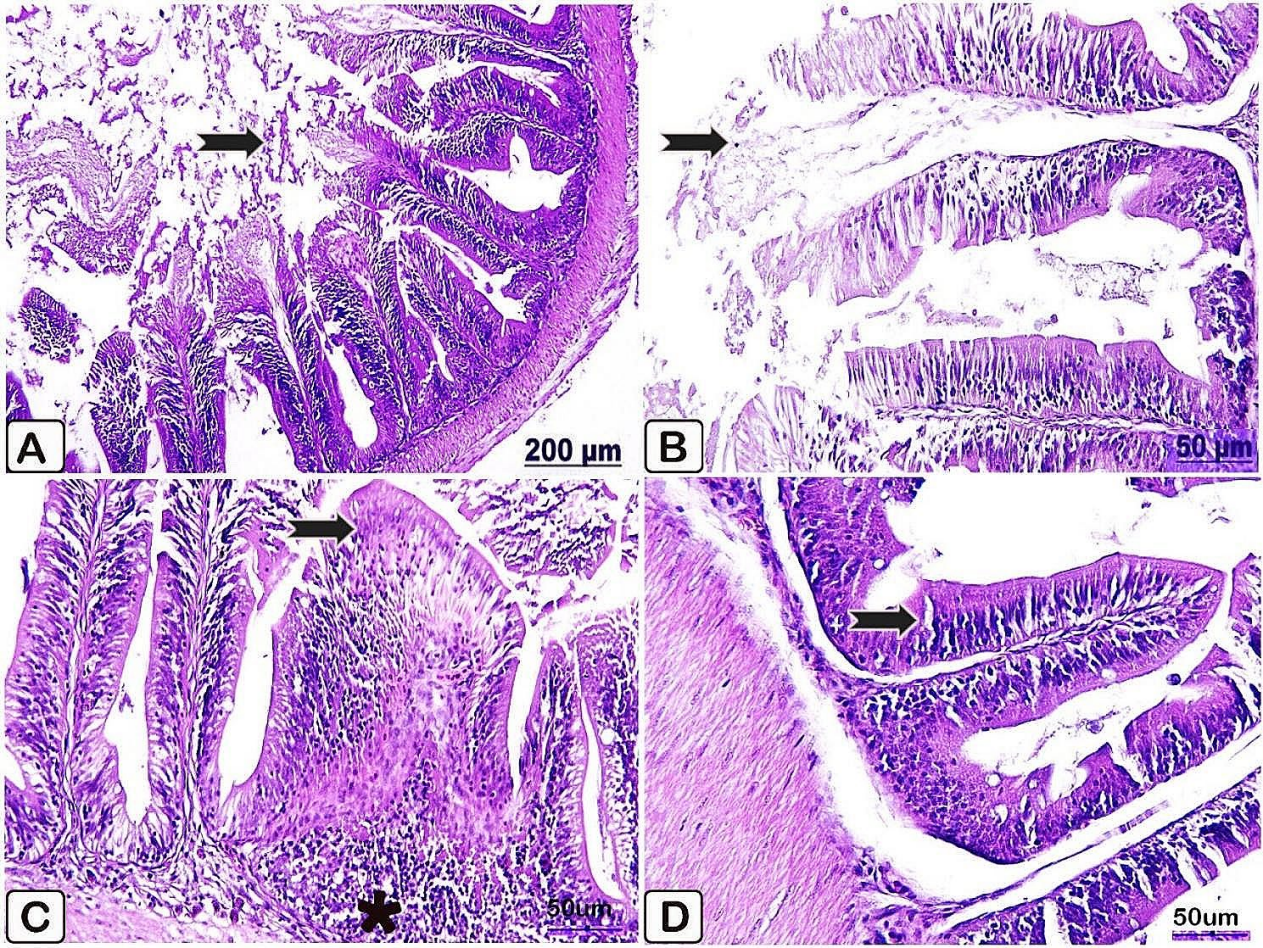
(**A**→**D**): Light paraffin microscopic sections with H & E stain from intestine of control Nile tilapia challenged with *P. putida* (**A, B**), intestine of Nile tilapia challenged supplemented group (**C, D**). (**A**) Light section showing necrosis with sloughing of the intestinal tips (arrow). (**B**) High power of Fig. A showing necrosis with sloughing of the tips of intestinal villi (arrow). (**C**) Light section showing intestinal epithelium lined with normal columnar cells (arrow), besides prominent accumulation of lymphocytes (star). (**D**) Light section showing apparent intact intestinal villi (arrow)

Control fish injected with *P. putida* exhibited significant histological aberrations in the target organs. Nevertheless, the challenged supplemented fish showed an improvement in histomorphological architectures, as detailed in Table 4; Figs. 9, 10, 11 and 12.

Microscopic examination of the control liver injected with *P. putida* displayed histological alterations represented by a remarkable degree of degenerative changes in the hepatocytes with cytoplasmic vacuolation and intensive congestion in the blood vessels (Fig. 9A, B). In contrast, the supplemented diet ameliorates the histological lesions that were detected in the control group, which manifested vacuolar degeneration of the hepatocytes and mild congestion in the blood sinusoids. A significant expression in the kupffer cells was observed in the challenge supplemented group when compared with challenge control group (Fig. 9C, D).

**Table 4:** Semi-quantitative histological scoring of the challenged control and supplemented fish

Groups Lesions	Control			Supplemented	
**Liver**					
Hepatocytes necrosis	+++			-	
Vacuolation of the hepatocytes	++			+	
Congestion in blood vessels	+++			+	
Leucocytes infiltration	++			+	
Activation of kpuffer cells	-			++	
**Anterior kidney**					
Necrosis of renal epithelium	++			-	
Degenerative changes of the renal tubules	++			+	
Melanomacrophages infiltration	+			+++	
Inflammatory cells infiltration	++			+	
Depletion in hematopoietic tissues	++			+	
Congestion of the blood vessels	+++			+	
**Spleen**					
Vacuolation and depletion of splenic white pulp		++		+	
Melanomacrophages infiltration		+		+++	
Hemorrhage and congestion of the red pulp		++		-	
Hemosiderosis		++		+	
**Intestine**					
Necrosis of the columnar epithelium		++		+	
Sloughing and desquamation of intestinal tips		+++		+	
Vacuolation of epithelial lining		++		++	
Lymphocytes infiltration		++		+++	
Stimulation of goblet cells		+		+++	
Absent, (-), mild (+), moderate (++), and severe (+++)					

Microscopic examination of the control anterior kidney injected with *P. putida* illustrated necrosis of the epithelial lining nephritic tubules, along with cytoplasmic vacuolation, and congestion in the blood vessels. Moderate depletion in the hematopoietic tissues was also seen (Fig. 10A, B). Compared to the challenged control group, healthy histological structures in the kidney of the challenged supplemented fish were observed, where they manifested intact nephritic tubules, and normal hematopoietic tissues (Fig. 10C, D).

Microscopic examination of the control spleen injected with *P. putida* showed histopathological lesions in white and red pulps, where red pulps suffered congestion of the hematopoietic tissues, and depletion of white pulp with minimally infiltrated melanomacrophages in the splenic tissues (Fig. 11A, B). In comparison with infected fish spleen, supplemented fish group spleen showed normal splenic pulps, healthy hematopoietic tissues, and massive aggregation of melanomacrophage centers (Fig. 11C, D).

Microscopic examination of the control intestine injected with *P. putida* manifested necrosis of epithelial lining intestinal villi with subsequent disintegration and desquamation of the intestinal tips (Fig. 12A, B). Histological analysis of the intestine of the supplemented fish group showed apparent normal criteria of the intestinal layers with pronounced infiltration of mononuclear cells mainly lymphocytes (Fig. 12C, D).

## Discussion

There is convincing evidence that herbal extracts stimulate the immune response and increase the disease resistance. Nevertheless, information concerning the stimulation and disease resistance of fish by the algae is lacking. Thus, our study aimed to investigate the effect of dietary supplementation with *P. boergesenii* water extract on the immunity and disease resistance of Nile tilapia.

The days of dietary supplementation for optimum induction of the immune response vary corresponding to the immunostimulant used. Herein, feeding Nile tilapia on a diet supplemented with *P. boergesenii* water extract for 20 successive days reflected a very non-specific immune response. The concurrent increase of both serum protein and globulin might be owed to the constant level of albumin. Elevations in serum protein and globulin concentrations are usually assumed to be correlated with a stronger innate immune response in fish [18] where globulins are created by the mononuclear phagocytes and they are critical components of the immune system.

These results coincide with those of Maghawri et al. [38], who found increases in total protein and globulin levels for the redbelly tilapia after using brown algae (*Padina pavonica*) as a feed additive. In a similar trend, previous studies in *Labeo rohita* after being fed diets supplemented with mixed algal extracts of *Euglena viridis, Chlorella vulgaris*, and *Spirulina platensis* [39] and diets supplemented with the seaweed, *Sargassum wightii*, and its fucoidan-rich extract [40].

The total antioxidant capacity is an important marker showing the state of the antioxidant function, including enzymatic and non-enzymatic antioxidants [41]. Our study revealed that dietary supplementation with *P. boergesenii* water extract for either 10 or 20 successive days significantly increased the TAC. These influences may be due to the structural properties of many secondary metabolites and bioactive compounds in *P. boergesenii* water extract, which have antioxidant activities that defend fish against oxidative injury, as confirmed by Oliveira et al. [15].

Interleukin-1β is an early-expressed proinflammatory cytokine that promotes the immune response by stimulating lymphocytes or generating the release of other cytokines that activate lymphocytes and macrophages [42]. The up-regulated *IL-1β* expression in our study confirms the immunostimulatory nature of the *P. boergesenii* water extract. Thus, dietary supplementation will clearly influence the gut mucosa after ingestion of the pellets. This site is a significant component of the mucosal immune system, that is one of the first barriers against fish infection [43], and hence this sensitization could lead to disease resistance. Also, the spleen, as the crucial immune organ, has been used as a marker of immune response in fish [44].

Our findings overlap with Watanuki et al. [45] who found increased *IL-1β* gene expression by administration of *Spirulina* sp. in common carp diets. Also, *IL-1* gene expression was associated with the feeding of common carp with polysaccharides from the *Padina gymnospora* [46]. Research reported that algae extract of *Capparis spinosa* improved the expression of *IL-1β* in the rainbow [47].

Moreover, this study showed up-regulation of *IL-1β* 2 days post-challenge with *P. putida* in the spleen and intestinal tissues, as they are the predilection sites of *P. putida*, indicating the stronger innate immune response of Nile tilapia. A recent study documented up-regulation of the *IL-1β* after bacterial challenge in *L. rohita* fed a diet comprising mixed algal extracts [39].

Antimicrobial peptides are important components of the innate immune system [26]. Therefore, expression studies of the AMPs may offer important data regarding the stimulation of their expression by immunostimulants and their contribution in protection against pathogens.

Only a limited number of studies related to AMPs expression following administration of immunostimulants, and few have demonstrated whether there is time effect on various AMPs in different tissues. This was the focus of the present study, which analyzed the expression of *β-defensin* and *NKL* in the intestine as a mucosal tissue and the spleen as a central immune tissue. This study showed that dietary supplementation with *P. boergesenii* water extract had a various effect on the different AMPs families in tissues. Such changes increase the antibacterial defenses at those tissues, and disease the bacterial resistance affected by the various AMPs families can be predicted. There are numerous causes that explain these changes in gene expression. For example, the expression levels of AMPs could relate to alterations in the leucocyte types in the tissues post-*P. boergesenii* administration or to alteration in their activation state. The expression of AMPs has been induced by pathogenic challenges [48, 49] as well as by immunostimulants in fish [26, 40].

Antimicrobial peptides have demonstrated their efficacy against gram-negative bacteria [50], including *P. putida*. Various AMP mechanisms have been examined, leading to the development of a model known as the Shai-Matsuzaki-Huang model. This model postulates that cationic AMPs can utilise their positive charge to attach to the anionic elements of the bacterial cell surface [21]. This attachment is suggested to cause killing of the bacterial cells through the leakage of bacterial cellular components.

Herein, this study is the first to record promoted immune-related gene expression levels in Nile tilapia fed a diet supplemented with *P. boergesenii* water extract. Therefore, this algae species can be listed as one of the immunostimulants that can modulate the expression of critical immune genes.

In recent years, there has been an increase in interest in algae extraction’s prophylactic abilities against bacterial challenges to aquatic animals. It has been stated that various algae extractions offer resistance to virulent bacteria *Aeromonas hydrophila* and *Edwardsiella tarda* in common carp [46], *A. hydrophila* in tilapia [51], *A. hydrophila* in rainbow trout [39] and to *P. aeruginosa* in *Cirrhinus mrigala* [16]. Nevertheless, in the literature, there is no study on the resistance of the *P. boergesenii* -fed Nile tilapia to *P. putida*.

In this study, a fish group fed on a diet supplemented with water extract of *P. boergesenii* showed a decrease in mortality rate and clinical signs, which could be attributed to the presence of different secondary metabolites such as flavonoids and fatty acids contents [15]. Where the GC-MS analysis of the volatile compounds of the marine algal extract showed that they have antibacterial [52, 53], and antioxidant activities [54, 55] that have a protective role in reducing the ulcerative injury induced by the *P. putida* toxins. Moreover, as confirmed herein, increased serum globulin may further promote resistance to infection. The findings suggested that the water extract of *P. boergesenii* has a promising potential immunostimulant, opening the door for the isolation of its active components.

The main lymphoid tissues are the liver, head kidney, spleen, and gastric mucosa in fish [56]. Accordingly, these tissues constitute the primitive analogues of fish lymph nodes for immune function [57]. According to our study, dietary inclusion of algae in aquafeed poses a synergistic effect on cell morphology with improved histoarchitectures, contrary to control fish. Hence, the histological observations on liver, kidney, spleen, and intestinal tissues confirm that the addition of *P. boergesenii* water extract to the fish feed exerts a histoprotective effect in Nile tilapia fish. These observations confirmed the protective impact of *P. boergesenii* water extract on the injured livers and other tissues induced by *P. putida* in the challenge experiment, permitting the alleviation of the histological deformations. This effect was probably mediated by the antioxidant and cytotoxic potential of the *P. boergesenii* water extract, owing to its flavonoids and fatty acid contents [15]. Algae-derived compounds could enhance the fish’s physiological function as well as change the histological damage [58].

Currently, at the histological level, *P. boergesenii* stimulates the cells that contribute to the immune response. Hence, the Kupffer cells were significantly activated in the liver sinusoids. Algae-derived bioactive ingredients, that can promote individual’s immune system, should be thought to raise the health condition of fish [59]. These findings are in parallel with Neyrinck et al. [60], who ensured that brown algae, *Laminaran*, when administered orally, could exert hepatoprotection. Herein, tilapia fed *P. boergesenii* water extract, followed by prominent activation of Melanomacrophage centers. Melanomacrophages serve as an applicable histological monitor of the immune response in fish [61]. Usage of spirulina (*Arthrospira platensis*) in feeding Nile tilapia displayed normal histomorphological structures in the liver, kidney, and spleen, with remarkable detection of melanomacrophage centers [62]. Also, the intestinal mucosa recorded proliferation of goblet cells and lymphocyte aggregation. A micro- and macroalgal blend could modulate the immune function of European seabass by supporting the migration of lymphocytes and monocytes to mucosal tissue and activating the proliferation of goblet cells in the intestine [59]. Similarly, Atlantic salmon, when fed an algae diet of *Schizochytrium* sp., resulted in a positive effect on salmon intestine health by enhancing intestinal mucus and goblet cell production [63]. Moreover, European seabass supplemented *Gracilaria gracilis* seaweed and *Nannochloropsis oceanica* microalga, which led to proliferation of goblet cells in the intestine [64].

## Conclusion

**Fig. 13:**
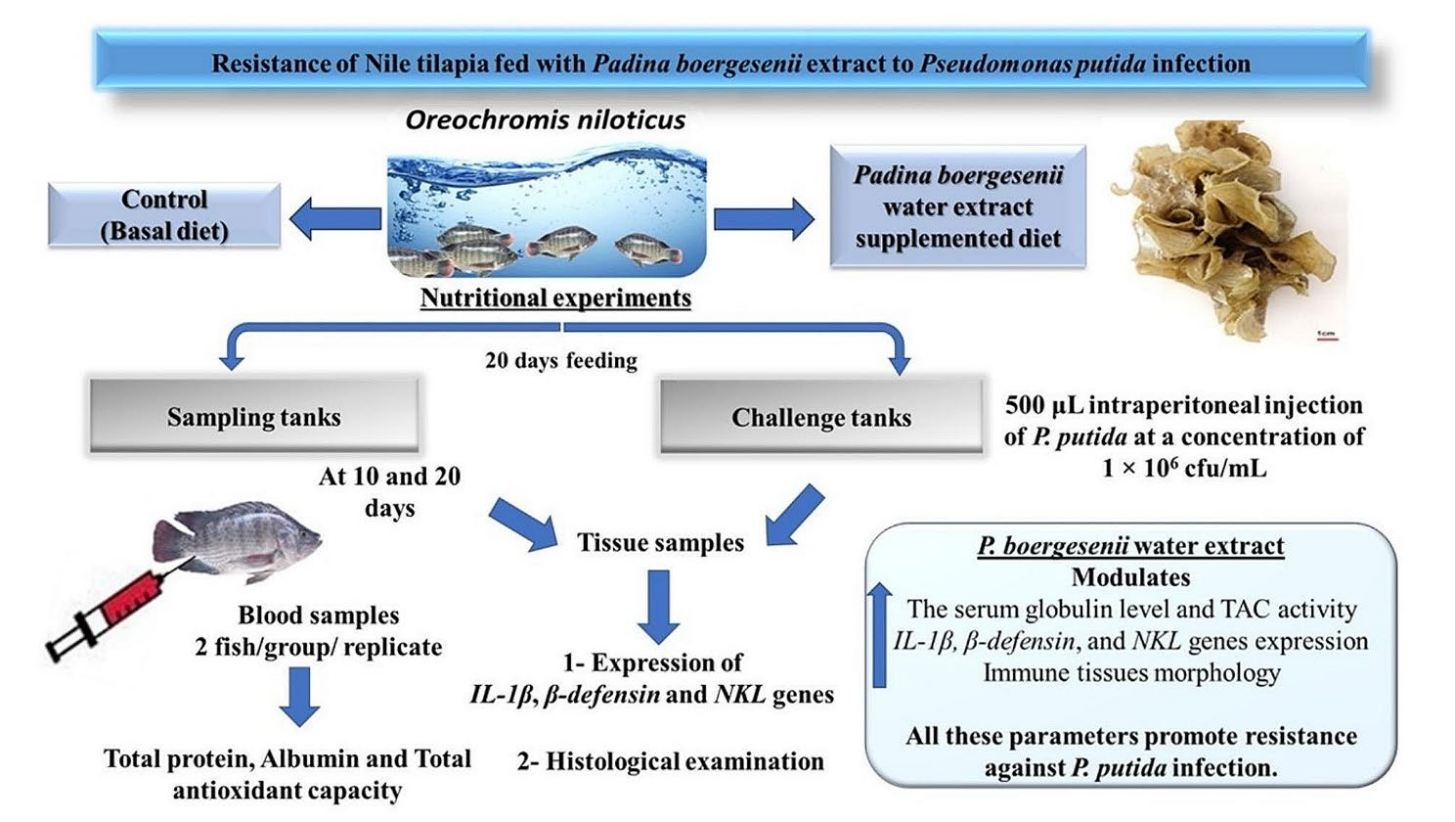
The experimental design and conclusion of the results

Feeding Nile tilapia with a diet supplemented with *P. boergesenii* water extract for 20 days significantly increased the serum globulin level and TAC activity and upregulated the expression of *IL-1β*, *β-defensin*, and *NKL* genes, along with their improvement to the cell morphology of the immune tissues, which all may promote resistance against *P. putida* infection. The experimental design and conclusion of the results are illustrated in Fig. 13.

## Data Availability

Requests for materials should be addressed to Karima A. Bakry and Walaa F.A. Emeish, Accession numbers: *P. putida* using 16s rDNA gene sequencing (GenBank accession no. OM048106, 838 bp).

## Abbreviations

*P. boergesenii*: *Padina boergesenii*
*O. niloticus*: *Oreochromis niloticus*
*P. putida*: *Pseudomonas putida*
GC-MS: Gas Chromatography Mass Spectrometry
ppt: Parts per thousand
TAC: Total antioxidant capacity
*IL-1β*: Interleukin-1beta
AMPs: Antimicrobial peptides
*β-defensin*: Beta-defensin
*NKl*: Natural killer-lysin
NK: Natural killer
CTLs: Cytotoxic T lymphocytes
cfu: Colony-forming unit
rDNA: Recombinant Deoxyribonucleic acid
RNA: Ribonucleic acid
Ct: Cycle threshold
H&E: Hematoxylin and eosin
SEM: Standard Error of Mean
ANOVA: Analysis of variance
RT: Retention time
A:G ratio: Albumin/globulin ratio
cDNA: Complementary Deoxyribonucleic acid
*β -actin*: Beta actin
*EF1α*: Elongation factor 1 alpha
qRT-PCR: Quantitative real-time Polymerase chain reaction
